# Impact of noncardiac findings in patients undergoing CT coronary angiography: a substudy of the Scottish computed tomography of the heart (SCOT-HEART) trial

**DOI:** 10.1007/s00330-017-5181-5

**Published:** 2018-01-02

**Authors:** Michelle C. Williams, Amanda Hunter, Anoop S. V. Shah, John Dreisbach, Jonathan R. Weir McCall, Mark T. Macmillan, Rachael Kirkbride, Fiona Hawke, Andrew Baird, Saeed Mirsadraee, Edwin J. R. van Beek, David E. Newby, Giles Roditi

**Affiliations:** 1 University of Edinburgh/British Heart Foundation Centre for Cardiovascular Science, Chancellor’s Building, 49 Little France Crescent, Edinburgh, EH16SUF UK; 2 Edinburgh Imaging facility QMRI, University of Edinburgh, Edinburgh, UK; 3 Department of Radiology, Glasgow Royal Infirmary, Glasgow, UK; 4 Division of Molecular and Clinical Medicine, University of Dundee, Dundee, UK; 5 Department of Radiology, Royal Infirmary of Edinburgh, Edinburgh, UK; 6 Department of Radiology, Borders General Hospital, Melrose, UK

**Keywords:** Computed tomography angiography, Coronary artery disease, Heart, Incidental findings, Lung neoplasms

## Abstract

**Objectives:**

Noncardiac findings are common on coronary computed tomography angiography (CCTA). We assessed the clinical impact of noncardiac findings, and potential changes to surveillance scans with the application of new lung nodule guidelines.

**Methods:**

This substudy of the SCOT-HEART randomized controlled trial assessed noncardiac findings identified on CCTA. Clinically significant noncardiac findings were those causing symptoms or requiring further investigation, follow-up or treatment. Lung nodule follow-up was undertaken following the 2005 Fleischner guidelines. The potential impact of the 2015 British Thoracic Society (BTS) and the 2017 Fleischner guidelines was assessed.

**Results:**

CCTA was performed in 1,778 patients and noncardiac findings were identified in 677 (38%). In 173 patients (10%) the abnormal findings were clinically significant and in 55 patients (3%) the findings were the cause of symptoms. Follow-up imaging was recommended in 136 patients (7.6%) and additional clinic consultations were organized in 46 patients (2.6%). Malignancy was diagnosed in 7 patients (0.4%). Application of the new lung nodule guidelines would have reduced the number of patients undergoing a follow-up CT scan: 68 fewer with the 2015 BTS guidelines and 78 fewer with the 2017 Fleischner guidelines; none of these patients subsequently developed malignancy.

**Conclusions:**

Clinically significant noncardiac findings are identified in 10% of patients undergoing CCTA. Application of new lung nodule guidelines will reduce the cost of surveillance, without the risk of missing malignancy.

**Key Points:**

*• Clinically significant noncardiac findings occur in 10% of patients undergoing CCTA.*

*• Noncardiac findings may be an important treatable cause of chest pain*

*• Further imaging investigations for noncardiac findings were recommended in 8% of patients after CCTA.*

*• New lung nodule follow-up guidelines will result in cost savings.*

## Introduction

The SCOT-HEART prospective multicentre randomized controlled trial showed that coronary computed tomography angiography (CCTA) in patients with suspected angina due to coronary heart disease improves diagnostic certainty, changes management and reduces future rates of myocardial infarction. [9] This has led to important changes in national guidelines which recommend the increased use of CCTA in patients with stable chest pain [1]. However, CCTA images visualize more than just the heart, and noncardiac findings can be an important cause of symptoms or require further investigation and management. With the increased use of CCTA, it is important to understand the downstream consequences of such noncardiac findings.

The follow-up of incidental lung nodules identified on computed tomography (CT) is dependent on local, national and international guidelines. In the SCOT-HEART trial, the 2005 Fleischner Society guidelines [2] were used to provide recommendations regarding lung nodule follow-up. These have recently been superseded by the 2015 British Thoracic Society (BTS) guidelines [3] and the 2017 Fleischner Society guidelines [4]. Trials of CT screening in patients at high risk of lung cancer have also established that CT of the chest can identify early lung cancers and reduce mortality in a cost-effective manner [5–7]. CCTA is often performed in patients with risk factors similar to those screened for lung cancer, such as smokers over the age of 55 years. Therefore, it is important to identify lung nodules on CCTA which may require further investigation and management.

In this substudy of the SCOT-HEART trial, we assessed the frequency and follow-up of noncardiac findings. In addition, we assessed the impact of changes in lung nodule follow-up guidelines on downstream investigations and costs.

## Materials and methods

### Study design and participants

The SCOT-HEART study was a multicentre randomized control trial of the use of CCTA in outpatients with suspected angina due to coronary artery disease [8]. The primary results of the SCOT-HEART study have been published [9]. Briefly, 4,146 patients who attended the Cardiology Outpatient Clinic were randomized to standard care or CT plus standard care, and followed up for symptoms, management and outcomes. Of these, 2,073 patients were randomized to CCTA, of whom 1,778 underwent CCTA. CCTA and non-contrast imaging for calcium scoring were performed as described previously [9], and this included a full field of view reconstruction of the chest in addition to CCTA reconstructions.

### Assessment of noncardiac findings

The presence of noncardiac findings was assessed on CCTA images focused on the heart, and also on the wide field of view images reconstructed to cover the entire scanned volume. In patients with partially imaged noncardiac findings identified at the time of scanning, a further full thoracic scan was not immediately performed, but could be subsequently recommended by the reporting radiologist. Images were reconstructed with standard soft tissue and lung reconstruction algorithms from each scanner. The reporting radiologist used soft tissue, lung and bone windowing parameters to view the images, with further manual adjustment as required.

Noncardiac findings were recorded by the reporting radiologist and the results were provided to the clinical team along with the CCTA results. Whether noncardiac findings were the cause of the patient’s symptoms was assessed on a four-point scale (‘yes’, ‘probable’, ‘unlikely’ or ‘no’). Clinically significant noncardiac findings were defined as those causing symptoms (‘yes’ or ‘probable’) or incidental findings requiring further investigation, follow-up or treatment.

### Assessment of lung nodules

Recommendations for lung nodule follow-up were provided to clinicians according to the 2005 Fleischner Society guidelines [2]. We assessed the potential change to the management of lung nodules if the 2015 BTS guidelines [3] and the 2017 Fleischner Society guidelines had been applied [4]. The lungs were assessed on wide field of view images reconstructed using a standard lung reconstruction algorithm. Lung nodule diameter was measured as the maximum diameter in any transverse projection, rounded to the nearest millimetre. Lung nodule volume was determined using Carestream Vue PACS (version 11; Carestream Health, Rochester, NY).

### Follow-up of noncardiac findings

Information on subsequent investigations for noncardiac findings were obtained from electronic health records. Information on clinic consultations were obtained from electronic health records or paper records where required. Imaging costs were obtained from the NHS Reference costs for 2014-2015 (Table 4) [10].

### Statistical analysis

Statistical analysis was performed using SPSS (version 23 for Mac OS X; IBM Corp., Armonk, NY). Normally distributed quantitative variables are presented as means and standard deviations. Non-normally distributed data are presented as medians and interquartile ranges (IQR). Statistical significance was assessed using Student’s *t* test, the Mann-Whitney U test or the chi-squared test as appropriate, and relative risks (RR) were calculated. A statistically significant difference was defined as a two-sided *p* value of <0.05.

## Results

### Noncardiac findings

Of the 1,778 patients who underwent CCTA, noncardiac findings were reported in 675 (38%). Patients with noncardiac findings were slightly older (60 ± 9 vs. 56 ± 9 years; *p* < 0.001) but there were no differences in gender, body mass index or presence of diabetes mellitus (Table 1). Patients who were current smokers or ex-smokers were more likely to have noncardiac findings than nonsmokers (RR 1.38, 95% confidence interval, CI, 1.22–1.56; *p* < 0.001). Patients with moderate or obstructive coronary artery disease were slightly more likely to have noncardiac findings than patients with mild disease or normal coronary arteries (RR 1.18, 95% CI 1.05–1.34; *p* = 0.005).

**Table 1:** Baseline patient characteristics

		Non-cardiac finding absent	Non-cardiac finding present	*p* value
Number of patients		1,103	675	
Age (years), mean ± SD		56 ± 9	59 ± 9	<0.001
Male, *n* (%)		626 (57)	372 (55)	0.522
BMI (kg/m^2^), mean ± SD		29.7 ± 5.7	29.5 ± 5.5	0.353
Smoking status, *n* (%)	Nonsmoker	580 (53)	268 (40)	<0.001
	Ex-smoker	324 (29)	271 (40)	
	Current smoker	199 (18)	135 (20)	
Diabetes mellitus, *n* (%)		128 (12)	68 (10)	0.349

**Fig. 1:**
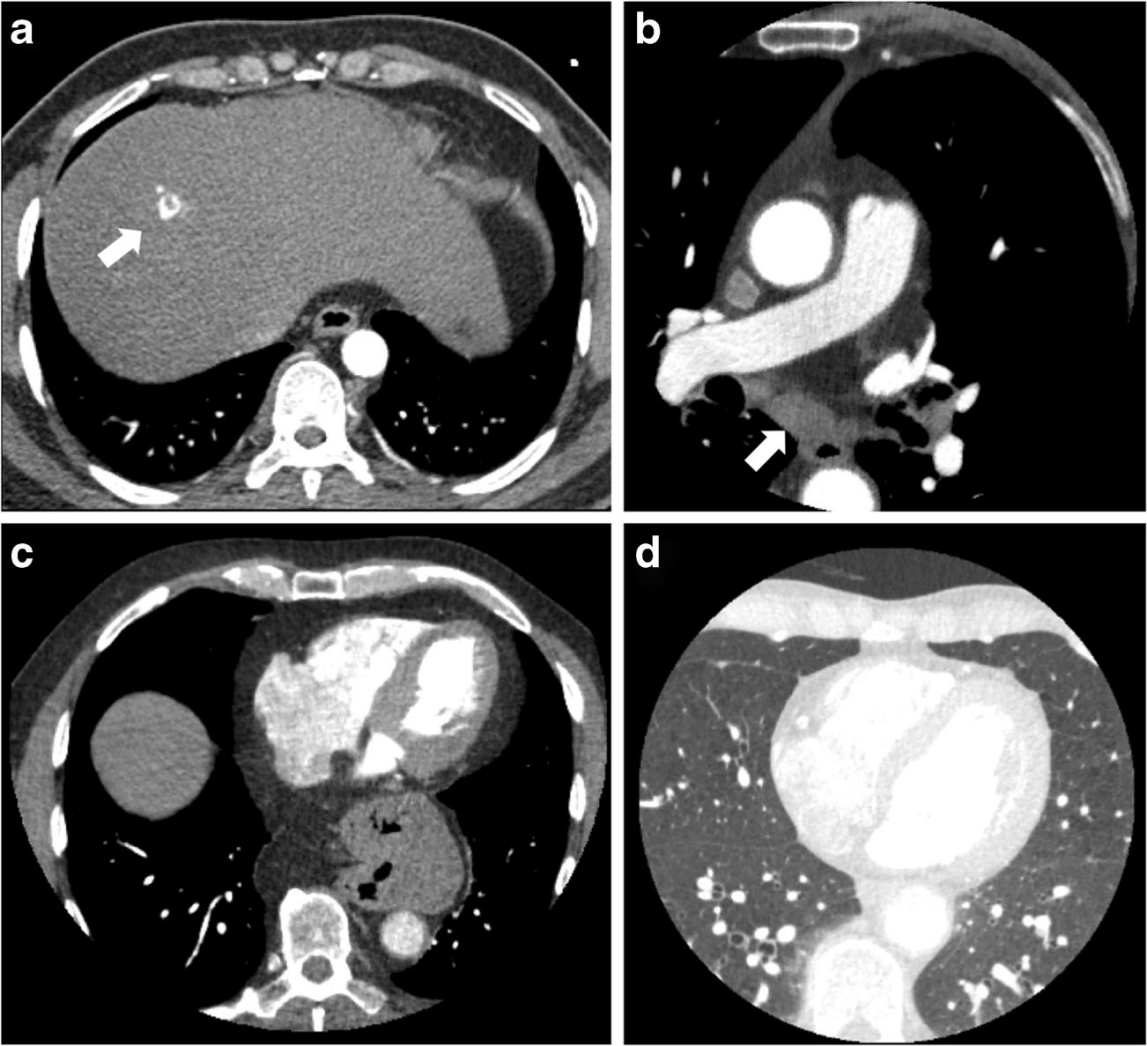
Examples of noncardiac findings identified on CCTA: a abnormal contrast enhancement in the liver (arrow) diagnosed as a benign haemangioma on subsequent imaging; b enlarged lymph node (arrow); c intrathoracic stomach; d centrilobular emphysema

**Fig. 2:**
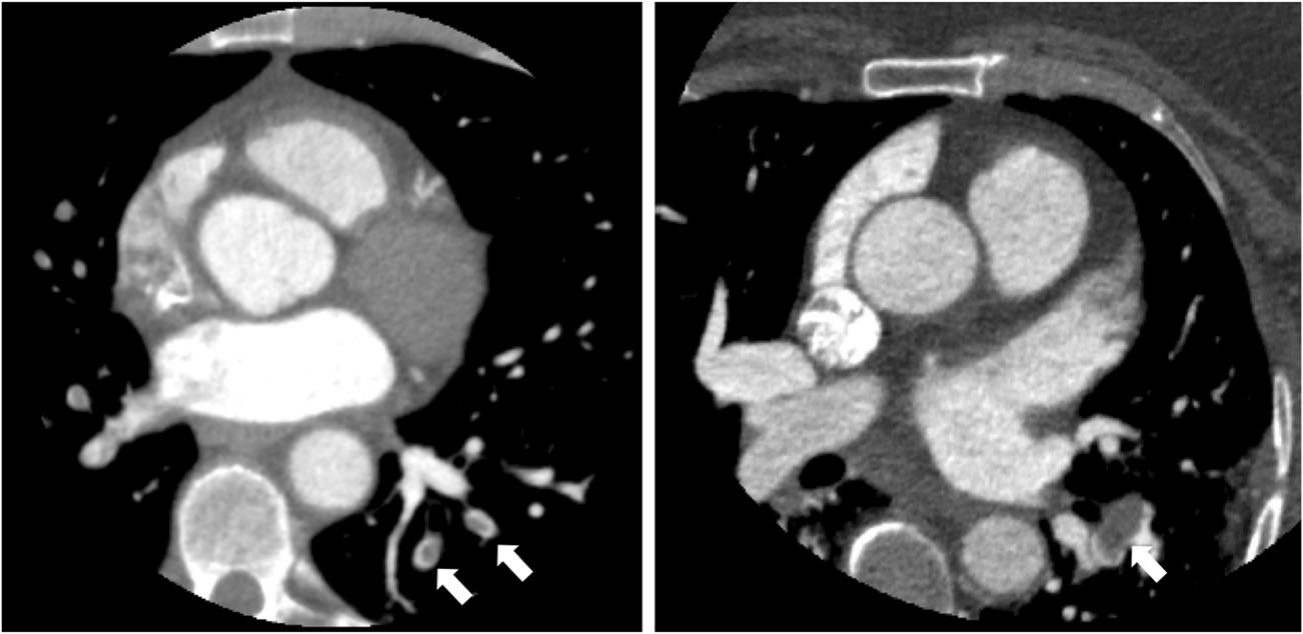
Examples of pulmonary emboli identified on CCTA in two patients (arrows)

In 175 of the 675 patients with noncardiac findings, the findings were defined as clinically significant (10% of all those undergoing CCTA, and 26% of those with noncardiac findings). There were no differences in age, gender, body mass index, presence of diabetes mellitus or presence of obstructive coronary artery disease between those with significant and those with nonsignificant noncardiac findings. The commonest findings were lung nodules or masses, emphysema and hiatus hernia (Table 2, Fig. 1). Noncardiac findings were deemed to be the definite cause of symptoms in 22 patients (1.2% of those undergoing CCTA, and 3.3% of those with noncardiac findings), and the probable cause of symptoms in a further 33 patients (1.9% and 4.9%, respectively). This included five patients (0.3% of those undergoing CCTA) with pulmonary emboli (Fig. 2).

**Table 2:** The frequency of noncardiac findings in patients undergoing CCTA for suspected angina due to coronary heart disease

System	Finding	Frequency
Lung	Emphysema or other parenchymal changes	202
	Lung mass/nodule/granuloma	200
	Atelectasis/scarring	63
	Pleural plaque	29
	Bronchiectasis	22
	Fibrosis	17
	Consolidation or pneumonia	15
	Pulmonary embolism	4
	Pleural effusion	3
Mediastinum	Lymphadenopathy	30
	Calcified lymph nodes	16
Aorta	Atheroma	26
	Dilation	17
Breast	Nodule	5
Liver	Cysts	36
	Haemangioma	2
	Fatty infiltration	2
Oesophagus	Hiatus hernia	121
	Thickening	3
Other	Anterior mediastinal mass, arteriovenous malformation, Bochdalek hernia, broncocele, duplication cyst, elevated left hemidiaphragm, gallstones, hamartoma, pericardial cyst, sclerotic vertebrae, syndesmophytes, subclavian vein stenosis, splenomegaly, splenic artery aneurysm, vertebral wedge fractures

### Investigation and management of noncardiac findings

Further imaging investigations for noncardiac findings were performed in 136 patients (7.6% of those undergoing CCTA, 20% of those with noncardiac findings). The most frequent follow-up imaging was CT for lung nodule assessment followed by ultrasonography of the liver and CT follow-up of other noncardiac findings (Table 3). Smokers were slightly more likely to undergo follow-up imaging (RR 1.41, 95% CI 1.02–1.96; *p* = 0.04), but there were no differences in rates of follow-up in relation to age, body-mass index, presence of diabetes mellitus or presence of obstructive coronary artery disease.

**Table 3:** Follow-up investigations and clinic consultations for noncardiac findings identified in patients undergoing CCTA

Follow-up investigations		Follow-up clinic consultations	
Investigation	Frequency	Clinic	Frequency
CT follow-up of other findings	12	Surgery	4
CT follow-up for lymphadenopathy	7	Oncology	3
PET/CT	4	Gastrointestinal	2
Chest plain radiography	6	Urology	2
Breast ultrasonography and mammography	4		
Liver MRI	2		
MRI for vascular assessment	1		
MRI for other findings	1		
Isotope bone scan	1		

Additional clinic consultations were organized in 46 patients (2.6% of those undergoing CCTA, 7% of those with noncardiac findings). The most frequent specialty to which patients were referred was the respiratory clinic (Table 3). There were no differences between patients who did or did not have follow-up in terms of age, body mass index, presence of diabetes mellitus, smoking status or presence of obstructive coronary artery disease.

Patients with respiratory infection, pulmonary embolism and malignancy identified on CCTA received appropriate therapy. In two patients with malignancy, the disease was too advanced at presentation and palliative management was given. The remaining patients underwent surgery (one patient), chemotherapy (one), chemotherapy and radiotherapy (one), pleurodesis (one), and chemoembolization (one). In contrast, there was no increase in gastric acid suppressant medication (RR 0.84, 95% CI 0.53–1.34; *p* = 0.466) in patients with a hiatus hernia nor were there demonstrable changes to treatment in patients with emphysema.

### Outcomes of patients with non-cardiac findings

**Fig. 3:**
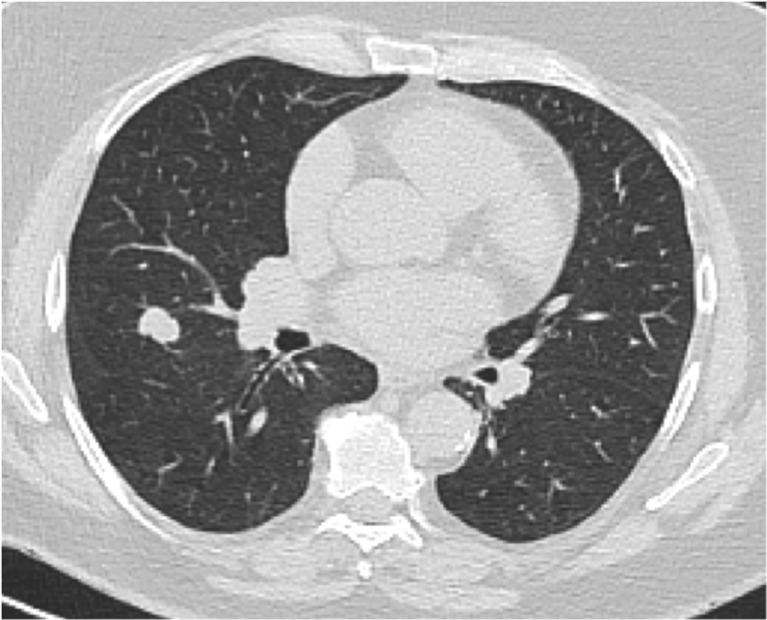
Example of lung cancer on a full field of view image

Malignancy was diagnosed in seven patients who underwent investigation for noncardiac findings (0.4% of patients undergoing CCTA, 3.5% of those with lung nodules). Malignancies included lung cancer (four patients) (Fig. 3), mesothelioma (one), metastatic testicular cancer (one) and hepatocellular carcinoma (one; Fig. 2). In patients with clinically significant noncardiac findings, there was no statistically significant difference in all-cause mortality (RR 2.50, 95% CI 0.70–8.87; *p* = 0.157).

### Lung nodules

Lung nodules, masses or granuloma were identified on CCTA in 200 patients (11% of those undergoing CCTA): ≤4 mm in 119 (60%), 5–7 mm in 58 (29%) and ≥8 mm in 23 (12%). Lung nodules were more common in current smokers or ex-smokers (RR 1.58, 95% CI 1.20–2.08; *p* = 0.001) but were not associated with age, gender, or presence of obstructive coronary artery disease. In 118 (59%) of the patients with lung nodules, semiautomated measurement of nodule volume was possible. The median lung nodule volume was 55 mm^3^ (IQR 32–116 mm^3^). Volume measurements were not possible in the remaining patients due to the small size of the lesions, proximity to structures of similar density, such as the pleura or diaphragm, or technical reasons.

Follow-up imaging for lung nodule assessment was recommended in 126 patients (7% of those undergoing CCTA). One patient died before follow-up imaging could be performed and 40 patients (20% of those with lung nodules) did not undergo follow-up imaging due to physician or patient choice. Thus 85 patients (4.7% of those undergoing CCTA) underwent CT follow-up for lung nodules. The median follow-up duration was 12 months (IQR 7–19 months) and the median number of CT scans performed was 1 (IQR 1–2). The first follow-up CT scan was performed at a median of 7 months after the initial CCTA scan (IQR 3.5–9.0 months), the second at 14 months (IQR 10.3–20.8 months), the third at 24 months (IQR 15–25.5 months) and the fifth at 31 months (IQR 26.8–35.3 months).

### Application of new lung nodule guidelines

The 2015 BTS guidelines suggest that no follow-up is required for lung nodules <5 mm or <80 mm^3^. In the SCOT-HEART study, this means that 68 fewer scans would have been performed in 47 patients, all of whom were subsequently discharged from follow-up without evidence of malignancy. This would have reduced the number of patients with significant lung nodules requiring follow-up to 38 (2% of patients undergoing CCTA). The 2017 Fleischner Society guidelines suggest that no follow-up is required for lung nodules of <6 mm or <100 mm^3^. In the SCOT-HEART study this means that 78 fewer scans would have been performed in 53 patients, all of whom were subsequently discharged from follow-up without evidence of malignancy. This would have reduced the number of patients with significant lung nodules requiring follow-up to 32 (2% of patients undergoing CCTA).

The cost of follow-up imaging for noncardiac findings was £10.06 per patient undergoing CCTA (1,778 patients, Table 4). The cost of imaging for lung nodule follow-up was £147.25 per patient undergoing lung nodule follow-up (85 patients) and £7.04 per patient undergoing CCTA (1,778 patients, Table 5). Applying the 2015 BTS guidelines would have reduced the cost per patient undergoing CCTA to £3.52 and applying the 2017 Fleischner Society guidelines would have reduced the cost to £3.00. This equates to 50% and 57% reductions in cost, respectively (Table 5).

**Table 4:** Cost (in UK pounds sterling) of imaging performed to investigate noncardiac findings in the 1,778 patients who underwent CCTA. Cost per scan was taken from NHS reference costs for 2014-2015 [10]

Investigation	Number of patients	Number of scans	Cost per scan (£)	Total cost per imaging modality (£)
CT chest for lung nodule follow-up	85	136	92.03	12,516.08
CT chest for other follow-up	10	11	104.07	1,144.77
CT chest, abdomen	1	1	120.92	120.92
CT chest, abdomen, pelvis	5	6	124.53	747.18
CT abdomen, pelvis	2	2	120.92	241.84
CT chest, neck	1	1	120.92	120.92
Liver ultrasonography	17	17	53.74	913.58
PET/CT	4	4	194.37	777.48
Chest plain radiography	6	6	30.00	180.00
Breast ultrasonography and mammography	4	4	53.74	214.96
Liver MRI	2	2	181.76	363.52
Other MRI	2	2	181.76	363.52
Isotope bone scan	1	1	188.77	188.77
Total cost of follow-up imaging (£)				17,893.54
Cost per patient for 1,778 patients undergoing CCTA (£)				10.06

**Table 5:** Cost of follow-up imaging for lung nodules and potential cost savings (in UK pounds sterling) of applying new lung nodule follow-up guidelines

	2005 Fleischner guidelines	2015 BTS guidelines	2017 Fleischner guidelines
Number of patients undergoing/predicted to undergo lung nodule follow-up	85	38	32
Number of CT scans required/predicted for lung nodule follow-up	136	68	58
Total cost of CT follow-up for lung nodules (£92.03 per CT) (£)	12,516.08	6,258.04	5,337.74
Cost per patient for 1,778 patients undergoing CCTA (£)	7.04	3.52	3.00

## Discussion

Although noncardiac findings on CCTA are common, only one quarter of these are clinically significant. Occasionally, these findings identify an important treatable cause of the patient’s chest pain but usually they represent important incidental findings that require surveillance, especially lung nodules. The application of new guidelines for lung nodule assessment will reduce the number of follow-up CT scans required in these patients, without the risk of missing malignancy identified during screening, and will significantly reduce the cost of follow-up imaging.

### Frequency and implications of noncardiac findings

The frequency of noncardiac findings in patients undergoing CCTA varies widely, and depends on the classification of findings and the population profile, such as age and frequency of smoking habit. A systematic review identified an average prevalence of 41% for noncardiac findings and 16% for clinically significant findings [11]. Our rates of noncardiac findings and significant findings were similar but slightly lower at 38% and 10%, respectively. This was possibly due to the wide inclusion criteria of the SCOT-HEART study, and the focus on a narrow detector range reducing both radiation dose and the scanned body volume.

Causes of chest pain can be challenging to diagnose. Patients in the SCOT-HEART trial were referred because of concern that they had angina pectoris due to coronary heart disease. In the CCTA group, ultimately 696 patients (33%) were diagnosed with this condition. In contrast, only a further 55 patients (3%) were diagnosed with alternative conditions that accounted for their symptoms including pneumonia, pulmonary embolism and cancer. This suggests a very modest rate of noncardiac causes of chest pain in this population. However, some of these noncardiac causes of chest pain are serious and potentially life-threatening, underlining the importance of full and comprehensive scan reporting including noncardiac structures.

### Lung nodule follow-up

The lungs were the commonest location of noncardiac findings in the SCOT-HEART study. This is similar to the findings of previous studies [11] and was largely driven by the prevalence of emphysema and lung nodules. The link between cardiovascular disease and respiratory diseases, such as emphysema, is complex and includes shared risk factors such as smoking and chronic inflammation [12, 13]. Lung cancer is the most frequent cause of death from cancer worldwide [5]. The National Lung Screening Trial (NLST) recruited asymptomatic participants aged 55 to 74 years, with a 30 pack-year smoking history and who were current smokers or had stopped smoking in the previous 15 years. The NLST identified a 20% (95% CI 6.8–26.7; *p* = 0.004) relative reduction in lung cancer mortality among 26,722 patients undergoing low-dose CT screening as compared with 26,732 patients undergoing plain radiography chest screening [14].

The NELSON study recruited 15,822 patients aged 50 to 75 years, who had smoked ≥15 cigarettes per day for ≥25 years or ≥10 cigarettes per day for ≥30 years and who were current smokers or had ceased smoking in the previous 10 years [15]. Participants were randomized 1:1 to CT screening or standard care. Early reports identified a beneficial effect of a screening interval of 2 years compared with 2.5 years [6], but at the time of this report the full trial results were awaited. The UK Lung Cancer Screening (UKLS) trial recruited 249,988 participants aged 50 to 75 years who had a ≥5% 5-year lung cancer risk based on the Liverpool Lung Project version 2 risk prediction model [7]. Participants were randomized 1:1 to CT screening or standard care. Pilot results showed that lung cancer could be identified at an early stage with potentially curative treatment possible in over 80% of patients [7]. However, at the time of this report the full results were also awaited.

The US Preventive Services Task Force recommends annual CT lung cancer screening in people aged 55 to 80 years, with a 30 pack-year history, and who were current smokers or had stopped smoking within the past 15 years [16]. The proportion of patients with lung nodules in the SCOT-HEART study was lower than in the lung cancer screening trials. This is probably partly due to the lower risk in the SCOT-HEART population, which included younger patients and nonsmokers. In addition, only part of the lungs are imaged on the wide field of view reconstruction from CCTA images as the scan range is selected to cover just the length of the heart rather than the full thorax. CCTA therefore does not image the upper parts of the lungs, a frequent location for lung cancer [17].

### New lung nodule follow-up guidelines

The new revised lung nodule follow-up guidelines have changed the threshold for lung nodules that require follow-up. In the 2015 BTS guidelines, no follow-up is required for “clearly benign lesions”, lesions <5 mm maximum diameter or lesions <80 mm^3^ volume [3]. In the 2017 Fleischner guidelines, no follow-up is required for single nodules <6 mm average diameter or <100 mm^3^ volume, but nodules with “suspicious” morphology or located in the upper lobe may be considered for a follow-up scan at 12 months [4]. Both new guidelines include the assessment of lung nodule volume rather than diameter as a potentially more reproducible method of assessment [4]. However, the value of automated lung nodule volume measurement is limited as only 59% of patients with lung nodules could be assessed in our study. In addition, whether maximum or average diameter is used may also affect the results. We retrospectively applied these diameter and volume criteria to the SCOT-HEART data which means we may have underestimated the benefit of the 2015 BTS guidelines and overestimated the benefit of the 2017 Fleischer guidelines. Nevertheless, we have shown that the application of these guidelines would lead to significant cost savings.

Previous studies have shown that the direct cost of imaging for significant incidental findings are between US$438 and US$606 [11, 18, 19]. The cost of follow-up imaging for significant noncardiac findings was lower in the SCOT-HEART study than in previous studies with a cost per patient of £10.06 averaged across all patients undergoing CCTA. However, there were significant differences in the healthcare systems involved in previous studies and therefore these figures are not directly comparable. There may also have been differences in the classification of significant noncardiac findings, in –the estimation of costs and in follow-up guidelines. In addition, the total cost of follow-up in the SCOT-HEART study should also include downstream clinic attendances and other nonimaging investigations which were not included in this estimate. The use of the national picture archiving system (PACS), that has been available in Scotland since 2008, for follow-up imaging means that any imaging performed in Scotland can be identified. However, a small number of patients may be missing if they have moved out of Scotland.

It is interesting to note that 20% of patients in whom subsequent imaging for lung nodule assessment was recommended did not undergo further imaging. This is a limitation of this study, but does highlight the real-world implications of the identification of noncardiac findings on CCTA. A variety of factors have been found to influence referral for further imaging including patient and physician factors [20].

## Conclusions

Significant noncardiac findings occur in 10% of patients undergoing CCTA for suspected angina due to coronary heart disease. Occasionally, these findings identify an important treatable cause of the patient’s chest pain but usually they represent important incidental findings that require surveillance, especially lung nodules. The application of new guidelines for lung nodule assessment will reduce the number of follow-up CT scans required in these patients, without the risk of missing malignancy identified during screening, and will significantly reduce the cost of follow-up imaging.
